# Long-Term Effects of Physical Exercise on Verbal Learning and Memory in Middle-Aged Adults: Results of a One-Year Follow-Up Study

**DOI:** 10.3390/brainsci2030332

**Published:** 2012-08-27

**Authors:** Kirsten Hötting, Gesche Schauenburg, Brigitte Röder

**Affiliations:** Biological Psychology and Neuropsychology, University of Hamburg, Hamburg 20146, Germany; Email: gesche.schauenburg@fu-berlin.de (G.S.); brigitte.roeder@uni-hamburg.de (B.R.)

**Keywords:** physical exercise, cardiovascular fitness, memory, attention, self-efficacy

## Abstract

A few months of physical exercise have been shown to increase cognition and to modulate brain functions in previously sedentary, mainly older adults. However, whether the preservation of newly gained cognitive capacities requires an active maintenance of the achieved fitness level during the intervention is not yet known. The aim of the present study was to test whether cardiovascular fitness one year after an exercise intervention was linked to cognitive variables. Twenty-five healthy participants (42-57 years of age) took part in a follow-up assessment one year after the end of a supervised exercise intervention. Measurements included a cardiovascular fitness test, psychometric tests of verbal learning and memory and selective attention as well as questionnaires assessing physical activity and self-efficacy beliefs. Recognition scores of participants with higher cardiovascular fitness at follow-up did not change significantly during the follow-up period; however, the scores of participants with lower cardiovascular fitness decreased. One year after the end of the physical training intervention, previously sedentary participants spent more hours exercising than prior to the intervention. The time participants spent exercising correlated with their self-efficacy beliefs. These results demonstrate a direct link between verbal learning and cardiovascular fitness and show that positive effects of physical interventions on learning and memory do need an active maintenance of cardiovascular fitness.

## 1. Introduction

Regular physical activity is associated with a reduced risk of chronic diseases like cardiovascular diseases, stroke, hypertension, type 2 diabetes mellitus, osteoporosis, obesity and some types of cancer [1]. Based on these findings, the American College of Sports Medicine and the American Heart Association recommend “moderate-intensity aerobic physical activity for a minimum of 30 min on five days a week or vigorous-intensity aerobic physical activity for a minimum of 20 min on three days a week” [1] to maintain health of adults between 18 and 65 years of age. However, less than half of the American adults meet these recommendations [1]; survey data from Germany suggested an even lower rate [2]. 

In addition to the prevention of chronic diseases, there is accumulating evidence from both animal and human studies that exercising has beneficial effects on the central nervous system and cognition [3]. Research in rodents has shown that running increases neurogenesis, enhances synaptic plasticity, spine density and brain vasculature, especially in the hippocampus [4]. Interventional studies on the effects of exercise on cognitive functions in humans have most often been conducted in aging populations. These studies suggest that previously sedentary older adults improve in tasks assessing executive functions [5,6], attention [7] and memory [8] after five to six months of regular exercise. Similar controlled studies in younger populations are rare so far and have provided more heterogeneous results. Stroth *et al*. [9], for example, reported improvements in visual-spatial memory for adults younger than 30 years of age after a six-week period of running training, but no significant change in verbal memory and attention. Pereira *et al*. [10] demonstrated improvements in short-term memory after 12 weeks of aerobic exercise in young adults (age range: 21–45 years) which correlated with an increase in cerebral blood flow in the dentate gyrus of the hippocampus. 

Recently, specific effects of an aerobic endurance training (cycling) and a non-endurance training (stretching/coordination) on verbal learning and attention, respectively, were reported in healthy adults between 40 and 56 years of age [11]: After six months of exercising, participants improved in verbal learning compared to a third, sedentary control group. The increase in the learning score correlated positively with the increase in cardiovascular fitness. Moreover, participants of the cycling group improved more in a delayed recognition test compared to both the stretching group and the sedentary control group. By contrast, attention was found to be mostly improved in the participants of the stretching/coordination training. 

Little is known about the sustainability of exercise induced effects on cognitive functions such as learning and memory. Prospective epidemiological studies have linked physical activity in middle-aged people to a reduced risk of dementia [12] and to larger gray matter density 20 years later [13]. To our knowledge, none of the published intervention studies targeting cognitive effects of physical exercise has reported a follow-up assessment. Usually, sedentary participants were recruited for studies on the effects of cardiovascular fitness on cognitive functioning. However, it is not known yet whether or how many of these participants continue exercising after the supervised training ended and what the consequences of post-intervention activity is with respect to cognitive variables. From a cognitive neuroscience point of view the expected post-intervention variance among previous research participants provides an opportunity to assess the link between cardiovascular fitness and cognitive function. From a public health point of view, it is important to know whether a few months of a supervised training might cause prevailing changes of the overall activity level and which factors modulate such lifestyle changes [14]. In social cognitive theory, efficacy beliefs (peoples’ belief in their capacities to execute an action) play a central role in predicting how long people sustain an action in the face of obstacles and failures [15]. Accordingly, self-efficacy beliefs have repeatedly been shown to predict the maintenance of physical activity (e.g., [16,17]). 

In this follow-up study, data is reported for participants who had taken part in a six-month long intervention of either an aerobic endurance training or a non-endurance training [11]. Physical activities and cardiovascular fitness were assessed one year after the end of the supervised training. Based on the finding of the initial intervention study that changes in cardiovascular fitness were related to the increase in verbal learning, it was hypothesized that cardiovascular fitness at follow-up was related to verbal learning and memory scores. Moreover, it was tested whether self-efficacy beliefs were related to physical activity levels. 

## 2. Methods

### 2.1. Study Design

**Figure 1:**
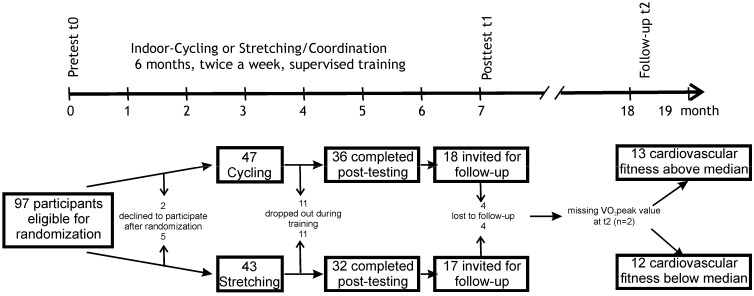
Study design and flow diagram showing the progress of participants through the phases of the study. Approximately half of the participants of the initial intervention study were assessed with magnetic resonance imaging (MRI) before and after the training. Only these participants were invited for a follow-up study one year after the end of the supervised training.

A summary of the study design is depicted in Figure 1. Details of the physical training and changes from the pretest (t0) to the posttest (t1) have been reported elsewhere [11]. In short: at the beginning of the study, participants were randomly assigned to either an aerobic endurance training (cycling) or a non-endurance training (stretching/coordination). Both groups exercised for 60 min twice a week for six months under the supervision of a qualified instructor. The intensity of the cycling training was adjusted to participants’ cardiovascular fitness as assessed at baseline (t0). The cycling training significantly increased the maximal oxygen consumption (VO_2_peak) from t0 to t1 (post-intervention assessment). The stretching/coordination training included stretching and toning of the whole body as well as exercises to improve coordination and flexibility. This group did not significantly improve their cardiovascular fitness from t0 to t1. At the end of the posttest, participants were encouraged to continue exercising and were informed about sports facilities in their neighborhood. There was no specific instruction to continue the type of exercise which they had been randomly assigned to. Participants were invited to a follow-up session (t2) one year after t1. Based on the results of a third cardiovascular fitness test at t2, they were divided into a low- *vs*. high-fit group. 

### 2.2. Participants

Participants of the present follow-up study were recruited from a sample that had taken part in a controlled intervention study (reported in [11]). Inclusion criteria for this initial study were a rather sedentary lifestyle (exercising less than twice a month). Exclusion criteria were a history of neurological diseases or any antidepressant or antipsychotic medications. Sixty-eight participants completed the initial study. Due to limited resources, only thirty-five of them were invited for a follow-up assessment one year after the end of the supervised training. These were the participants who had been assigned to be additionally assessed with MRI. This group did not differ in training compliance, in cardiovascular fitness or in cognitive variables at either t0 or t1 from the remaining participants (all *p* > 0.1). Six participants did not take part in the follow-up assessment due to a lack of time, one participant dropped out due to illness and one participant had died in an accident. Due to technical problems, cardiovascular fitness data was missing for two participants at t2. Thus, 25 complete datasets were available at t2. Mean age of these participants was 51 years (SD = 4.2, range 42–57 years), 15 of them were female. Six subjects had a middle school diploma (equivalent to the German “Hauptschule”, grade 9 or “Realschule”, grade 10) and had completed their apprenticeship; three had A level certificates as well as an apprenticeship qualification. Sixteen had an university degree. During the time of the intervention study, most of the subjects worked in their qualified profession. One subject was unemployed, two where full-time homemakers. Thirteen participants of the follow-up sample had taken part in the cycling training and twelve had participated in the stretching/coordination training between t0 and t1. Participants’ characteristics were summarized in Table 1. 

The study was conducted in accordance with the ethical standards laid down in the declaration of Helsinki and was approved by the ethical board of the German Psychological Society (DGPs). All participants had given informed consent. 

**Table 1:** Participants’ characteristics separated by participants with high cardiovascular fitness *vs*. low cardiovascular fitness at t2 and drop-outs.

	High fit at t2 ( *n* = 13)	Low fit at t2 ( *n* = 12)	Drop-outs ( *n* = 10)	*p* (high fit *vs.* low fit *vs.* drop-outs)	*p* (high fit *vs.* low fit)
Age at t0 (mean, SD)	48.15 (3.69)	50.25 (4.55)	48.60 (3.50)	0.40 ^a^	0.22 ^c^
Sex (female/male)	8/5	7/5	4/6	0.56 ^b^	0.87 ^b^
Vocabulary score ^1^ (mean, SD)	31.77 (3.94)	33.25 (2.45)	32.60 (1.58)	0.46 ^a^	0.28 ^c^
Years of education (mean, SD)	13.69 (2.18)	13.33 (2.35)	14.00 (2.11)	0.78 ^a^	0.70 ^c^
Body mass index at t1(mean, SD)	25.41 (3.33)	26.43 (4.31)	26.82 (3.19)	0.63 ^a^	0.51 ^c^
Time between t1 and t2 in days (mean, SD)	353.69 (13.87)	349.33 (18.62)			0.51 ^c^
Training group during intervention phase (cycling/stretching)	8/5	5/7	5/5	0.61 ^b^	0.32 ^b^
Number of training sessions during intervention phase (mean, SD)	46.46 (7.99)	48.50 (2.32)	45.70 (7.53)	0.57 ^a^	0.40 ^c^

^a^ ANOVA; ^b^ Chi-square test; ^c^ *t*-test; ^1^ Assessed with the multiple choice vocabulary test [18]. This test allows a good estimate of the global IQ in healthy adults and is especially sensitive to the educational background.

### 2.3. Assessments

Measurements of physical activity and cognition had been taken at baseline (t0), after six months of a supervised physical training (t1) and were again recorded at the follow-up one year after the end of the intervention (t2). Questionnaires on self-efficiency were administered at t2 only.

#### 2.3.1. Cardiovascular Fitness and Physical Activity

Cardiovascular fitness was assessed by a standardized stepwise incremental cycle ergometer test [19]. The peak of the oxygen uptake volume (VO_2_peak) was used as the indicator of cardiovascular fitness. During the test, heart rate and respiration were continuously measured with an ergospirometric measuring station (MetaMax I, Cortex, Leipzig, Germany). Oxygen and carbon dioxide concentrations in the breath were analyzed and served to determine VO_2_peak (MetaSoft 1.11.5, Cortex, Leipzig, Germany). The initial workload depended on the participant’s physical constitution and was 25 W or 50 W. Depending on the initial value, workload was increased by a total of 25 W (8.3 W per minute) or 50 W (16.7 W per minute), respectively, over the course of three minutes. The incremental exercise test was terminated when subjective exhaustion was reached. Due to technical problems, the VO_2_peak data were missing for two participants at t2. Thus, all analyses based on VO_2_peak values at t2 included 25 participants only. 

Participants’ general physical activity was assessed with the “Freiburger Questionnaire for Physical Activity” [20]. This questionnaire comprises 12 items about basic physical activity (e.g., walking to work, taking the stairs, gardening) as well as leisure-time activities (e.g., dancing) and sports activities (regularly performed activities to maintain or improve physical fitness). Participants were asked to indicate how much time they spent with these activities during the last week or month. For data analysis, all scores were converted into hours per week. The total duration of physical activity (sum of all items) and the duration of sports activities are reported in the following. The retest-reliability after six months for both scores has been reported to be *r* = 0.56 [20]. Validation studies have shown moderate positive correlations between self-reported physical activity duration and VO_2_peak measurements [20]. 

#### 2.3.2. Cognitive Variables

Cognitive variables were selected based on the results of the initial intervention study. These results showed an association between verbal learning and cardiovascular fitness and an effect of the stretching/coordination training on attention [11]. 

The German equivalent of the Auditory Verbal Learning Test was used to assess verbal learning [21]. A list of 15 words was read five times by the experimenter and the participants were asked to memorize and to recall as many words as possible after each presentation. The learning score is defined as the sum of correctly recalled words across all five presentations. After 30 min, a delayed recognition test was administered. The recognition score was calculated as the difference between correctly detected old words minus false alarms to new words. The retest reliability for both scores ranges from *r* = 0.79 to *r* = 0.82 [21]. The Auditory Verbal Learning Test shows moderate correlations to other tests of learning and memory like the California Verbal Learning Test or the Benton-Test and is sensitive to memory deficits in patients with temporal lobe dysfunctions and beginning Alzheimer’s disease [21]. 

Selective attention was assessed with the “d2” test [22]. During this paper-and-pencil letter cancelation task, participants were asked to mark all letters “d” tagged with two dashes among distractors. They processed 14 rows of 47 letters each under time pressure (20 s per row). The attention score was defined as the number of correctly detected targets minus the number of false alarms. The attention score has a high internal consistency (α = 0.95) and retest reliability (*r* > 0.80) [22]. The test score shows moderate correlations to Go/NoGo tasks, trail making tests and selective attention tasks [22]. 

#### 2.3.3. Self-Efficacy

Perceived general self-efficacy and self-efficacy for physical exercise were assessed with two questionnaires: (1) The general perceived self-efficacy scale [23] comprises ten items on how participants cope with different life events (e.g., “I can always manage to solve difficult problems if I try hard enough”). Internal consistencies for this scale are between α = 0.75 and α = 0.92 and retest-reliability ranges between *r* = 0.47 and *r* = 0.67 [24]. Convergent and discriminant validity has been demonstrated by positive correlations to self-esteem, optimism, coping and self-regulation and negative correlations to anxiety, depression and procrastination [24,25]. (2) The physical exercise self-efficacy scale [26] comprises 20 items and asks how confident participants are to overcome barriers that may interfere with regular exercising (e.g., “I can manage to carry out my exercise intentions, even when I am tired”). Responses were given on a 4-point scale. Reliability and validity studies on a short version of this scale have reported an internal consistency of α = 0.89, positive correlations to the intention towards physical exercise and negative correlations to psychological distress and physical discomfort [27]. Moreover, the scale has been shown to discriminate between physically active and inactive individuals [27]. Sum scores were calculated separately for general self-efficacy and physical exercise self-efficacy. 

### 2.4. Data Analysis

First, changes in cardiovascular and cognitive variables between t1 and t2 were compared between participants who took part in the cycling *vs*. stretching training during the intervention phase. Therefore, analyses of variance (ANOVA) with Time (t1 *vs*. t2) as within-participant factor and Group (cycling *vs*. stretching/coordination) as between-participant factor were run. 

Second, it was tested whether changes in cognitive variables were modulated by the cardiovascular fitness level at follow-up. Based on the VO_2_peak score at t2, participants were divided into a “high-fit group” and a “low-fit group” using a median split procedure (median of all participants; using separate medians for men and women resulted in the same group assignments). To test whether cognitive variables and the duration of physical activities had changed differently for these two groups from t0 (baseline) to t1 (after the end of the training intervention) and to t2 (follow-up) analyses of covariance (ANCOVA) with Time (t0 *vs*. t1 *vs*. t2) as within-participant factor, Fitness (high fit *vs*. low fit) as between-participant factor and age, sex, years of education and training group during the intervention phase as covariates were run. Whenever the Time × Fitness interaction reached significance level (*p* < 0.05), changes across time were analyzed separately for each group with paired *t-*tests. 

Associations between self-efficacy and cardiovascular fitness or self-reported physical activity at t2 were calculated with partial correlations controlling for age, verbal-IQ and cardiovascular fitness or physical activity at t1, respectively.

## 3. Results

Mean cardiovascular fitness across all participants decreased from 32.72 mL/min/kg (SE: 1.01) at t1 to 30.15 mL/min/kg (SE: 0.91) at follow-up (*t*(22) = 2.13, *p* = 0.045). VO_2_peak scores are separately depicted for the cycling *vs*. stretching group for all time points in Table 2. Cardiovascular fitness tended to decrease in both intervention groups from t1 to t2 with no significant difference between groups (main effect of Time *F*(1,21) = 4.08, *p* = 0.056, η^2^ =0.16; Time × Group *F*(1,21) = 0.14, *p* = 0.71, η^2^ = 0.01). There was a significant overall decrease in verbal learning from t1 to t2 as well (main effect of Time *F*(1,25) = 4.49, *p* = 0.044, η^2^ = 0.15 for the recognition score; *F*(1,25) = 47.18, *p* < 0.001, η^2^ = 0.65 for the learning score). The decrease in the learning score tended to be more pronounced for the stretching group compared to the cycling group (*F*(1,25) = 3.82, *p* = 0.062, η^2^ = 0.13) while there were no differences between groups for the recognition score (Time × Group *F*(1,25) = 0.12, *p* = 0.73, η^2^ = 0.01). Both groups showed a trend towards an increase in the attention score from t1 to t2 (main effect Time *F*(1,25) = 3.52, *p* = 0.07, η^2^ = 0.12, Time × Group *F*(1,25) = 0.10, *p* = 0.75, η^2^ < 0.01). 

**Table 2:** Cardiovascular fitness and cognitive variables separately for participants of the cycling training *vs*. stretching training (mean with standard deviation in parenthesis).

		t0	t1	t2	*p*
VO_2_peak	Cycling	27.79 (5.00)	32.89 (6.14)	29.92 (4.46)	0.71
	Stretching	30.33 (3.91)	32.49 (2.66)	31.28 (6.64)	
Recognition score after 30 min	Cycling	13.21 (1.76)	14.07 (1.21)	13.00 (3.19)	0.73
	Stretching	13.69 (1.89)	14.69 (0.63)	13.92 (1.56)	
Learning score	Cycling	59.29 (6.60)	62.71 (7.35)	58.00 (7.90)	0.06
	Stretching	58.23 (7.36)	66.54 (4.39)	58.08 (5.94)	
Attention score	Cycling	154.79 (34.19)	164.50 (41.65)	168.43 (39.58)	0.75
	Stretching	163.08 (24.34)	179.69 (29.73)	185.23 (33.80)	

Note: *p*-values for the Group (cycling *vs*. stretching) × Time (t1 *vs*. t2) interaction.

To test whether changes in cognitive variables across time depended on participants’ cardiovascular fitness one year after the intervention period, cognitive data were analyzed with regard to the cardiovascular fitness level at t2 irrespective of original group assignment. As seen in Table 1, the groups generated by the median split on VO_2_peak data did not differ significantly in age, sex, verbal-IQ, body-mass index, number of attended training sessions during intervention and the time passed between the end of the intervention and follow-up. Moreover, the number of participants who took part in the cycling *vs*. stretching training during the intervention phase did not differ significantly between the high *vs*. low fit group. Drop-outs did not differ from the studied participants with regard to these variables either (all *p* > 0.2). Moreover, the low *vs*. high fit groups studied at t2 did not differ in any of the cardiovascular or cognitive outcome variables at baseline (t0) or after the intervention (t1, all *p* > 0.1).

**Figure 2:**
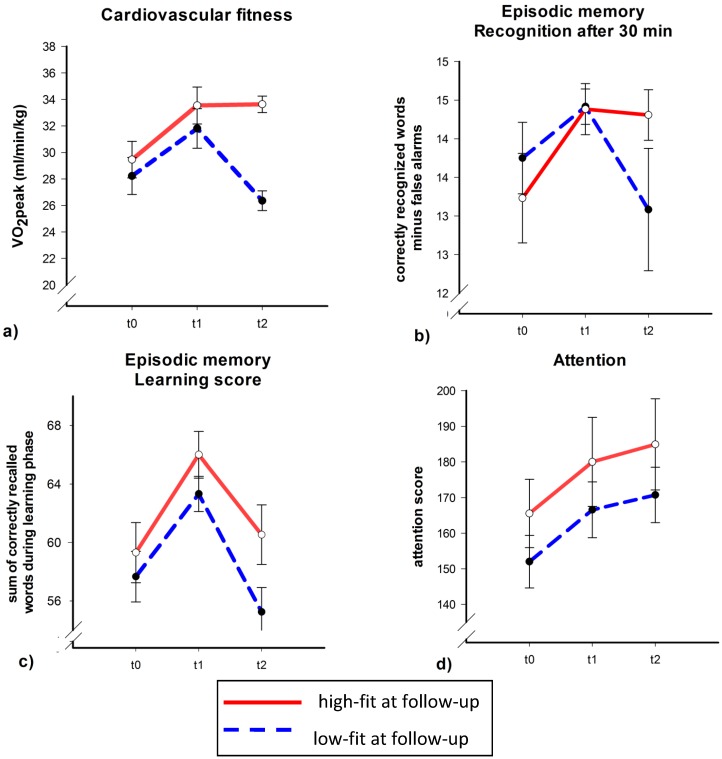
Cardiovascular fitness (**a**), verbal learning (recognition score (**b**) and learning score (**c**)), and attention scores (**d**) separately for participants with high fitness (red, solid line) *vs*. low fitness (blue, dashed line) at follow-up. Mean with standard error bars.

As depicted in Figure 2a, both the high and low fit group (at t2) had gained in cardiovascular fitness from t0 to t1. The high fit group, however, did not show any significant change in VO_2_peak between t1 and t2 while cardiovascular fitness decreased in the low fit group during this time period. This was confirmed by a significant Time × Fitness interaction (*F*(2,34) = 3.34, *p* = 0.048, η^2^ = 0.16). Follow-up analyses showed a significant or a marginal significant increase in VO_2_peak from t0 to t1 in both the high and low fit group (*t*(11) = 3.37, *p* = 0.006 for the high fit group and *t*(10) = 2.05, *p* = 0.068 for the low fit group). From t1 to t2 only the low fit group had lost overall fitness (low fit group: *t*(10) = −4.00, *p* = 0.003; high fit group; *t*(11) = 0.06, *p* = 0.96).

To test whether participants with high *vs*. low cardiovascular fitness at t2 differ in verbal learning capacities, we run similar ANCOVAs with verbal learning and recognition scores as dependent variables. The changes in recognition after a delay of 30 min mirrored the changes in cardiovascular fitness (Figure 2b): recognition scores of participants with high cardiovascular fitness at t2 did not change from t1 to t2 (*t*(12) = −0.23, *p* = 0.82), while scores of participants with low cardiovascular fitness at t2 tended to decrease (*t*(11) = −1.94, *p* = 0.079). Importantly, the Time × Fitness interaction was significant (*F*(2,38) = 3.42, *p* = 0.043, η^2^ = 0.15). Although the learning score seemed to increase in both fitness groups from t0 to t1 and decreased from t1 to t2 (Figure 2c), the ANCOVA controlling for age, sex, years of education and training group during intervention, did not indicate a significant main effect of Time or Time × Group interaction (all *p* > 0.3). 

By contrast, selective attention as assessed by the d2 seemed to increase monotonously across the three measurement times irrespective of cardiovascular fitness (Figure 2d). But neither the main effect of Time nor the Time × Group interaction reached significance in the ANCOVA (all *p* > 0.2). 

**Figure 3:**
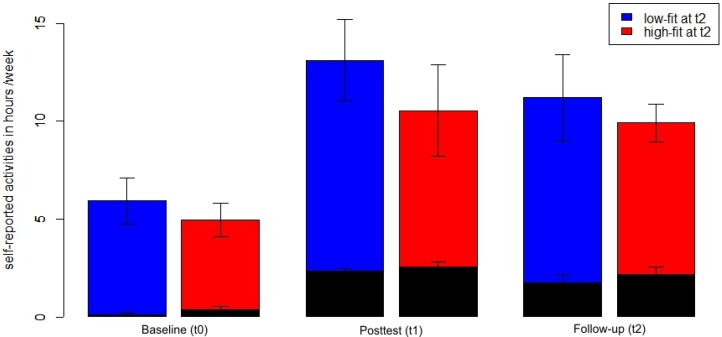
Total physical activity (blue and red bars) and sports activities (black, superimposed bars) in hours per week across time points separately for participants with high fitness (red) *vs*. low fitness (blue) at t2. Mean with standard error bars.

Participants with high *vs*. low cardiovascular fitness at t2, however, did not report a different duration of physical activity during a week (Figure 3). There was neither a significant main effect of Fitness nor any Time × Fitness interaction for the time at sports activities or the total duration of physical activity assessed by the physical activity questionnaire (all *F* < 1.4, all *p* > 0.25, η^2^ < 0.06). 

During the time of the study, participants increased both the time they spent exercising (main effect Time *F*(2,46) = 37.06, *p* < 0.001, η^2^ = 0.62 for the time at sports activities) and their overall physical activity (main effect Time *F*(2,46) = 11.24, *p* < 0.001, η^2^ = 0.33 for the total duration of physical activity). The total physical activity score represents the sum of sports activities, physical activities in the daily routine and leisure time activities with low intensity (e.g., bowling and dancing). Thus, during the time of the study participants established a more physically active lifestyle. One year after the end of the supervised training, participants were able to maintain this active lifestyle: there was only a slight, non-significant mean decrease of sports activities and total amount of physical activities from t1 to t2 (*t*(24) = 1.57, *p* = 0.13 and *t*(24) = 0.86, *p* = 0.40, respectively). Both scores at t2 were significantly higher than at t0 (both *t*(24) > 3.8, *p* < 0.01). 

Participants’ physical exercise self-efficacy correlated with the hours of sports activities they reported in the physical activity questionnaire at t2: the higher participants’ physical exercise self-efficacy, the more hours of sports activities they reported (*r* = 0.54, *p* = 0.009, after adjustment for age, verbal IQ and sports activities at t1). Physical exercise self-efficacy did neither significantly correlate with the hours of self-reported total physical activity (*r* = 0.22, *p* = 0.324) nor with VO_2_peak (*r* = 0.04, *p* = 0.858) at t2. General self-efficacy was not associated with hours of sports activities, total physical activity or cardiovascular fitness (all *r* < 0.3, all *p* > 0.2).

## 4. Discussion

This study used a one year follow-up of participants who had participated in a six-month physical exercising intervention (cycling *vs*. stretching) to test whether cognitive gains achieved during the intervention phase were maintained and whether this maintenance depended on the cardiovascular fitness at follow-up. During the one-year follow-up, verbal learning decreased within both groups with a trend for the learning score to decrease more in the stretching group compared to the cycling group. This decrease, however, was modulated by cardiovascular fitness at follow-up: participants with higher cardiovascular fitness at follow-up did not change in memory while there was a decrease for participants with lower cardiovascular fitness. In addition, we found that participants maintained their overall increased amount of physical activity and sports activities one year after the supervised training ended; the time they exercised during a week was a function of reported self-efficiency beliefs. 

Animal studies have suggested that the hippocampus, a core structure for inducing the consolidation of long-term memories [28], is especially sensitive to exercise [29] and thus, may contribute to exercise induced changes in learning and memory [30]. In humans, the volume of the hippocampus has been shown to correlate with cardiovascular fitness [31] and an aerobic exercise intervention seems to counteract age-related hippocampal volume loss [32]. At the behavioral level, better memory performance has been reported after exercising both in older [8,32]; and in young adults [9,10]. The question whether an increase in cardiovascular fitness is necessary for exercise induced increases in memory, however, is still a matter of debate. The studies by Erickson *et al*. [32] and Ruschewey *et al*. [8] reported improvements in memory after both an aerobic exercise training and a low-intensity exercise training (gymnastics/stretching) and no significant group differences in changes from pre- to posttest. The results of an intervention study in middle-aged adults are partially in line with these reports: both an aerobic cycling training and a stretching/coordination training led to better learning in middle-aged adults compared to a sedentary control group [11]. The improvement in verbal learning in this study, however, correlated positively with VO_2_peak across all groups suggesting that changes in cardiovascular fitness predict parts of the variance in memory after the training (see also [10] for a similar relationship).

The most important finding of the present follow-up study is that cardiovascular fitness seems to be a modulator of delayed recognition of verbal material in the long run. Only participants with a cardiovascular fitness level above the group median one year after the end of a supervised training maintained their enhanced recognition performance. By contrast, participants with lower cardiovascular fitness lost in memory performance. These results suggest that enhancing cardiovascular fitness might prevent age related memory decline in the long run. 

Only recognition of verbally learned items after a delay of 30 min but not immediate memory (learning score of the auditory verbal learning test) or attention were found to be a function of cardiovascular fitness. It could be speculated that in middle-aged people, long-term memory might be the first and thus most sensitive function to gain from cardiovascular fitness. Long-term memory starts to decline already in the 20’s [33] and a shrinkage of medial temporal lobe structures has been reported even in healthy, middle-aged adults [34]. However, the selectivity needs to be further proved compared to other cognitive domains. 

Since cardiovascular fitness explains only a small amount of variance in cognitive variables, other factors have to be considered in future studies to explain the interdependence between physical exercise and cognitive functioning. Inconsistent findings with regard to the link between cardiovascular fitness and cognitive variables across studies might be due to the rather unspecific effect of cardiovascular fitness on brain functions [35]. Assessing biological markers of brain plasticity like the brain-derived neurotrophic factor (BDNF), insulin-like growth factor I (IGF-1) or granulocyte colony stimulating factor (G-CSF) might allow characterizing the link between exercising and cognition more precisely. Moreover, different kinds of exercise might induce differential effects on cognition and behavior, possibly via distinct mechanisms on the neurophysiologic level [36]. Due to the small sample size in the actual study, it was not possible to split groups with respect to the kind of exercise they choose during the follow-up phase. 

Although not all participants were able to maintain the cardiovascular fitness level achieved after the end of the supervised training, taking part in six months exercise training increased both general physical activity and sports activities in adults who were rather sedentary before entering the study. On average, participants reported exercising 0.25 h/week at baseline and 2 h at follow-up. The high fit group and low fit group did not differ in the time they reported exercising at follow-up suggesting that both groups were able to establish a more active lifestyle. The VO_2_peak difference between groups might suggest that the high fit group choose more intense activities compared to the low fit group. Alternative explanations, however, could be individual differences in participants’ responsiveness to aerobic activity or in individual differences in the habituation speed to a constant level of physical activities. The American College of Sports Medicine recommends doing moderate-intensity exercise at least 30 min on five days a week to maintain general health [1]. Seventy-four percent of this sample continued exercising on average for more than 2.5 h a week. Thus, one year after a supervised training, most of the previously sedentary participants now met these recommendations. Relative to aerobic fitness norms for this age group, however, only the VO_2_peak values of the high fit group were average at t2 while cardiovascular fitness of the low fit participants was still partially far below average [37]. Data on the long-term maintenance of physical activity after structured exercise training are rare so far, especially in healthy, middle-aged adults. Compared to results of counseling programs that aimed to enhance physical activity [38,39], the maintenance rate of the present sample was quite high. This could be due to the recruitment procedure of the sample: probably only sedentary people who were highly motivated to increase their physical activities responded to the advertisement of the present study. 

The main goal of the present study was to analyze long-term effects of exercising on cognition and links between cognition and cardiovascular fitness. Nevertheless, it is worth thinking about what contributes to this successful implementation of a physically active lifestyle in previously sedentary adults. Self-efficacy beliefs have been identified as an important predictor of the maintenance of physical activity [17]. In line with these results, a positive correlation between exercising and physical exercise self-efficacy was found in the present study as well. However, it is not possible to derive causal relations from this correlation. Since self-efficacy was assessed at t2 only, it is not possible to distinguish whether self-efficacy influences exercise maintenance or whether the experience of being able to maintain the activity level led to higher self-efficacy beliefs. Structural equation models have shown that the frequency of previous exercise influences self-efficacy beliefs and thus, these beliefs then influence the maintenance of exercising in the future [17]. Moreover, according to this model, social support in exercise groups and positive affects are associated with the maintenance of a higher physical exercise level mediated by self-efficacy beliefs. It might be speculated that the highly structured and supervised exercise training in the intervention phase of the present study initiated similar mechanisms as described by McAuley *et al*. [17]: sedentary adults received social support from their exercise group, they may have experienced positive affects after the training and they learned that they are able to exercise on a regular basis. This experience then increased self-efficacy beliefs about exercising and thus, contributed to a long-term maintenance. Since these are yet pure speculations, the role of self-efficacy and possibly other personal traits for enhancing the attendance rate in physical exercising programs needs to be systematically tested. If known, parallel interventions addressing these variables might enhance overall benefits of such programs.

Some shortcomings of the present study need to be discussed as well. The sample size was rather small and it was only possible to invite about half of the original sample at follow-up. However, since there were no systematic differences between the invited participants and not invited participants with respect to cognitive and cardiovascular variables, we consider the reported results as representative for the intervention sample. Nevertheless, larger long-term studies are desirable to confirm these first results.

From a public health point of view, the present results are promising as they demonstrate that six months of exercising has sustainable effects on physical activity and cognition in previously sedentary adults beyond a supervised training period. Cardiovascular fitness seems to contribute to the beneficial effects of exercise on memory. Thus, attempts should be made to encourage physical activities in healthy middle-aged adults. In the long run, establishing a physically active lifestyle might reduce age-related cognitive decline, both by enhancing neuronal plasticity and reducing the risk for chronic diseases that were associated with cognitive decline [3,14]. 
